# Infrared Thermography for Simultaneous Detection of Limb Pathology and Subclinical Mastitis in Dairy Cows

**DOI:** 10.3390/ani16132060

**Published:** 2026-07-03

**Authors:** Juozas Žemaitis, Ugnė Spancernienė, Vaida Jokubauskienė, Ignas Šilinskas, Kristina Musayeva, Rasa Želvytė, Judita Žymantienė, Antanas Sederevičius, Joris Vėžys, Vytautas Jūrėnas, Algimantas Bubulis, Sigitas Japertas, Vytautas Ostaševičius, Vaidas Oberauskas

**Affiliations:** 1 Department of Anatomy and Physiology, Faculty of Veterinary Medicine, Lithuanian University of Health Sciences, 47181 Kaunas, Lithuania; ugne.spancerniene@lsmu.lt (U.S.); vaida.jokubauskiene@lsmu.lt (V.J.); kristina.musayeva@lsmu.lt (K.M.); rasa.zelvyte@lsmu.lt (R.Ž.); judita.zymantiene@lsmu.lt (J.Ž.); 2 Research Centre for Digestive Physiology and Pathology, Department of Anatomy and Physiology, Faculty of Veterinary Medicine, Lithuanian University of Health Sciences, 47181 Kaunas, Lithuania; ignas.silinskas@lsmu.lt (I.Š.); antanas.sederevicius@lsmu.lt (A.S.); 3 Department of Mechanical Engineering, Mechanical Engineering and Design Faculty, Kaunas University of Technology, 51424 Kaunas, Lithuania; joris.vezys@ktu.lt; 4 Institute of Mechatronics, Kaunas University of Technology, 51424 Kaunas, Lithuania; vytautas.jurenas@ktu.lt (V.J.); algimantas.bubulis@ktu.lt (A.B.); vytautas.ostasevicius@ktu.lt (V.O.); 5 Practical Training and Testing Centre, Lithuanian University of Health Sciences, Akacijų St. 2, 54310 Giraitė, Lithuania; sigitas.japertas@lsmu.lt

**Keywords:** cattle, infrared thermal imaging, lameness, mastitis, screening, precision livestock farming

## Abstract

This study evaluated infrared thermography (IRT) as a dual-purpose, non-invasive tool for detecting limb pathology and subclinical mastitis in dairy cows. Surface temperatures were measured at the coffin, pastern, and fetlock regions of all limbs and at the teat surface of all udder quarters. Limb health status was determined by orthopedic clinical examination, and mastitis status was assessed using the California Mastitis Test (CMT). Hindlimbs were significantly warmer than forelimbs in healthy cows (*p* < 0.001), with confirmed bilateral symmetry (r = 0.524–0.569). Limbs with pathology showed significantly higher temperatures at all anatomical sites, with particularly large effect sizes in the hindlimbs (r around 0.80). Additional lesion-specific analyses demonstrated that both infectious and claw horn lesions exhibited significantly higher temperatures than healthy limbs. In the hindlimbs, infectious lesions showed higher thermographic values than claw horn lesions. CMT-positive quarters showed teat temperatures of 30.0–30.2 °C compared with 25.2–25.8 °C in CMT-negative quarters (*p* < 0.001). Receiver operating characteristic (ROC) analysis demonstrated excellent diagnostic performance for teat surface temperature in identifying CMT-positive quarters, with an area under the ROC curve (AUC) of 0.956. The optimal threshold was 29.5 °C, corresponding to a sensitivity of 0.992 and a specificity of 0.838. Each 1 °C increase in teat temperature increased the odds of CMT positivity by 5.3-fold (*p* < 0.001). A practical three-zone thermographic framework was established: <27 °C (healthy), 27–29.5 °C (monitoring recommended), and ≥29.5 °C (mastitis likely). Teat temperature was not associated with composite milk somatic cell count, but hindlimb pathology was independently associated with elevated teat temperatures (partial r = 0.23–0.26, *p* ≤ 0.001). These findings demonstrate that IRT has strong potential as an integrated screening tool for dairy cow limb health and udder health monitoring.

## Simple Summary

Lameness and mastitis are among the most important health disorders in dairy farming because they impair animal welfare, reduce productivity, increase treatment and culling costs. Early detection remains challenging under commercial conditions, particularly when routine examination of individual animals is time-consuming and dependent on subjective clinical assessment. Infrared thermography is a non-invasive imaging technique that measures surface temperature and can detect inflammatory changes before obvious clinical signs become visible. In this study, thermographic images of the limbs and teats of dairy cows were compared with orthopedic findings and California Mastitis Test results (CMT). Cows with limb pathology exhibited significantly higher limb surface temperatures than unaffected cows, and CMT-positive quarters had teat temperatures that were approximately 5 °C higher than CMT-negative quarters. Both infectious and claw horn-related lesions were associated with increased limb temperatures, and infectious hindlimb lesions exhibited the highest thermographic values. A practical three-zone classification system was developed for mastitis screening: <27 °C indicated a low probability of mastitis, 27–29.5 °C indicated the need for monitoring or confirmatory testing, and ≥29.5 °C indicated a high probability of mastitis. Hindlimb pathology was also associated with elevated teat temperatures, suggesting a possible biological link between locomotor disorders and udder health. These findings indicate that a single thermographic examination may support simultaneous screening for both limb disorders and subclinical mastitis in dairy cows.

## 1. Introduction

Lameness and mastitis remain the two most prevalent and economically important health disorders in dairy cattle, causing substantial losses through reduced milk yield, impaired reproductive performance, increased treatment costs, and premature culling [1,2]. In addition to their economic burden, both conditions represent major animal welfare concerns, particularly lameness, which is associated with pain, altered behaviour, and impaired mobility [1,3]. The global incidence of lameness affects 13–50% of dairy cows, with estimated costs of USD 337 per case and annual losses exceeding USD 6 billion worldwide [1,4]. Subclinical mastitis is similarly problematic because it often progresses in the absence of visible clinical signs while persistently reducing both milk yield and milk quality [5,6]. Early and reliable detection of these conditions remains difficult under typical commercial dairy farm conditions [3,7]. Conventional lameness assessment, particularly locomotion scoring, is widely used but is inherently subjective, dependent on observer experience, and often insufficiently sensitive for detecting early-stage pathology [7,8]. Similarly, mastitis screening methods such as the California Mastitis Test (CMT) and somatic cell count evaluation require repeated sampling, animal handling, and, in some cases, laboratory support, which may limit their suitability for frequent or large-scale herd monitoring [6,9]. These constraints have intensified interest in precision livestock farming technologies capable of delivering objective, rapid, and non-invasive health assessment at the individual-animal level [3,10].

Infrared thermography (IRT) has emerged as a promising approach in this context because it enables contactless assessment of surface temperature distribution and may reveal physiological disturbances associated with inflammation before overt clinical signs become apparent [11,12]. Infrared thermography detects infrared radiation emitted from the body surface and converts it into thermal images that reflect local blood perfusion, tissue metabolism, and heat dissipation [11,12]. Its diagnostic rationale is based on the inflammatory process, during which vasodilation, increased blood flow, and enhanced tissue metabolism elevate local surface temperature in affected regions [12,13]. Because these thermal changes may precede visible anatomical or behavioural abnormalities, IRT has considerable potential as an early-warning tool in dairy herd health monitoring [10,13]. In studies of bovine lameness, IRT has been used to assess hoof and distal limb temperatures, and pathological limbs have consistently demonstrated higher surface temperatures compared with healthy limbs [1,4,7,13]. Previous work has also demonstrated that baseline thermal patterns vary according to limb position, with hindlimbs generally displaying higher temperatures than forelimbs, highlighting the need for anatomical standardization and limb-specific interpretation [1,4]. In parallel, IRT has been investigated as a diagnostic aid for mastitis, and udder quarters affected by intramammary inflammation have repeatedly shown higher surface temperatures relative to healthy quarters [5,14,15]. Recent evidence, including machine learning-based studies, further suggests that thermographic data may support automated and accurate classification of mastitis status, although diagnostic accuracy remains strongly dependent on imaging protocol, anatomical measurement site, environmental conditions, and the selected reference standard [5,10,16].

Despite this progress, the practical application of IRT in dairy herd health management remains limited by several unresolved methodological and biological issues [10,12]. First, most published studies have addressed lameness and mastitis separately, and only limited attention has been given to the possibility of evaluating limb and udder thermography simultaneously within the same animals [1,5,12]. This restricts assessment of whether a single IRT session could serve as a dual-purpose screening tool under farm conditions [3,12]. Second, clinical and epidemiological evidence indicates that lameness, particularly hindlimb disorders, may increase mastitis risk through altered lying behaviour, reduced mobility, poorer hygiene, and greater exposure of the teats to environmental pathogens; however, this relationship has not been systematically investigated using thermographic indicators [17,18]. Third, although several thermal thresholds for mastitis detection have been proposed, a standardized and practically interpretable classification framework for on-farm use is still lacking [5,12,16]. Finally, limited attention has been paid to the relationship between quarter-level teat surface temperature and composite milk composition parameters. Moreover, the potential effects of comparing quarter-level thermographic measurements with cow-level composite milk indicators have not been adequately investigated [9].

Recent advances in data-driven analytics further strengthen the relevance of this field [10]. Machine learning approaches applied to thermographic data have shown promising performance for automated cattle health assessment, suggesting that IRT could become an important component of future precision livestock systems [10,16]. Based on these considerations, the present study was designed to test the hypothesis that infrared thermography can function as an integrated, non-invasive tool for the simultaneous detection of limb pathology and subclinical mastitis in dairy cows, and that hindlimb pathology may be associated with altered udder thermal patterns. Therefore, the objectives of this study were (1) to evaluate the diagnostic capacity of limb surface temperature measured by IRT for detecting forelimb and hindlimb pathology in dairy cows; (2) to assess the diagnostic performance of teat surface temperature for subclinical mastitis detection using the California Mastitis Test as the reference method; (3) to investigate the relationship between teat surface temperature and composite milk composition parameters; and (4) to examine the association between limb pathology and udder thermal patterns. An additional objective was to propose a practical thermographic classification framework for on-farm mastitis screening.

## 2. Materials and Methods

### 2.1. Animals

A total of 105 lactating Holstein–Friesian dairy cows housed at the Practical Training and Testing Centre of the Lithuanian University of Health Sciences (Giraitė, Lithuania) were initially evaluated for inclusion in this cross-sectional study. Cows were included irrespective of parity and days in milk (DIM). Individual parity and DIM data were retrieved from the DeLaval DelPro herd management software (version 5.12; DeLaval International AB, Tumba, Sweden) and used as covariates in the mixed-effects logistic regression model. Animals were housed in a free-stall barn equipped with automatic waterers, ventilators, a scraper-based manure removal system, and dry bedding supplemented with zeolite. Milking was performed using a DeLaval robotic milking system, and cows were fed a total mixed ration twice daily with ad libitum access to water. The study was conducted in October 2025.

Thermographic image quality assessment was performed prior to data analysis. Eight cows were excluded because thermographic images of either the udder or distal limb regions did not meet predefined image quality criteria due to visible contamination or excessive moisture. Consequently, 97 clinically healthy cows, defined as animals without overt systemic clinical signs at the time of examination, were included in the final analyses.

All procedures involving animals were performed in accordance with institutional ethical standards and were approved by the Bioethics Committee of the Lithuanian University of Health Sciences (protocol No. 2024-BEC3-T-006, 12 March 2024). Written informed consent was obtained from the owner of the animals. All procedures were non-invasive and did not involve induction of disease, painful interventions, or experimental treatments. Animal handling was minimized to reduce stress during examinations and thermographic imaging.

### 2.2. Experimental Design

**Figure 1:**
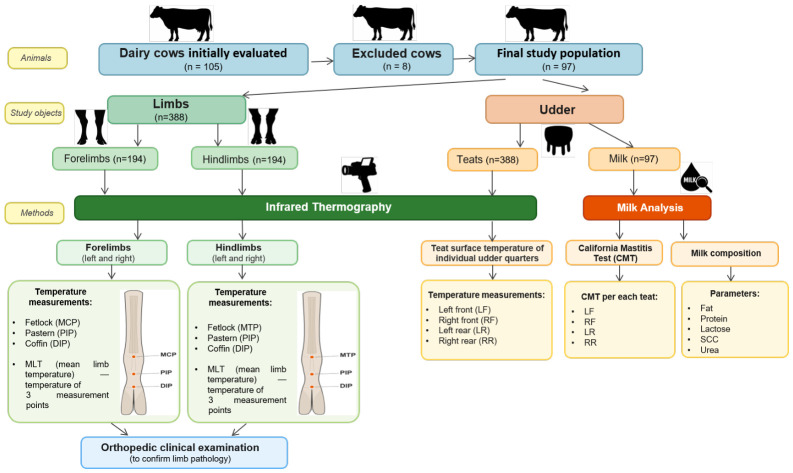
Flowchart of animal enrollment, thermographic image quality assessment, and analytical workflow used in the study. A total of 105 cows were initially evaluated, and eight cows were excluded because thermographic images of the udder or distal limbs did not meet predefined image quality criteria due to visible contamination or excessive moisture. Final analyses were performed using data from 97 cows.

Figure 1 presents an overview of the experimental design, including animal enrollment, image quality assessment, exclusions, and the final number of cows and thermographic image sets included at each analytical stage. Two primary study objects were evaluated in each cow: (i) limbs (forelimbs and hindlimbs) and (ii) udder (teats and milk). All examinations, including infrared thermographic imaging, the California Mastitis Test (CMT), milk sample collection, and orthopedic clinical examination, were carried out on the same day for each animal. The workflow was standardized as follows: after entry into the milking parlour, thermographic images of the limbs and udder were obtained; the CMT was then performed immediately before milking; milk samples were collected during milking for composition analysis; and orthopedic examination of all limbs was conducted after milking.

### 2.3. Infrared Thermography

#### 2.3.1. Equipment and Image Acquisition

Infrared thermographic images were acquired using a FLIR T640 camera (FLIR Systems Inc., Wilsonville, OR, USA; 640 × 480 pixels; thermal sensitivity <0.03 °C at 30 °C; accuracy ±2 °C). The thermal camera emissivity setting was adjusted to 0.98, which is appropriate for biological tissues. Imaging was performed indoors when cows entered the milking parlour under controlled environmental conditions (ambient temperature approximately 15 °C, no direct solar radiation, and no detectable air drafts). Although obvious sources of air movement and skin contamination were avoided and images with visible moisture or dirt were excluded from analysis, relative humidity, air velocity and skin dryness were not quantitatively recorded or experimentally fixed. The camera was positioned approximately 1.0 m from the target surface and maintained perpendicular to the anatomical area being evaluated. Images were captured under consistent lighting conditions to minimize reflective artefacts. For each cow, thermographic images were obtained from the dorsal aspect of the forelimbs and hindlimbs and from the udder with all four teats visible in a single frame. Thermal images were analyzed using FLIR Tools software (version 5.1.15036.1001; FLIR Systems Inc., Wilsonville, OR, USA). Images affected by substantial contamination, excessive moisture, movement artefacts, or incomplete visualization of the evaluated anatomical region were excluded from analysis. Thermographic image quality assessment was performed prior to statistical analysis. Overall, eight cows were excluded because thermographic images of either the udder or distal limb regions did not meet predefined image quality criteria. Imaging was performed at a similar time of day to minimize circadian variation in surface temperature measurements.

#### 2.3.2. Limb Thermography

Thermographic images were obtained from all four limbs (both forelimbs and both hindlimbs) of each cow. Skin surface temperature (°C) was measured at three anatomical reference points on the dorsal aspect of the distal limb.

For the forelimbs (metacarpal region), the measurement points were defined as follows:

- metacarpophalangeal (MCP) joint—fetlock;
- metacarpal proximal interphalangeal (PIP) joint—pastern;
- metacarpal distal interphalangeal (DIP) joint—coffin.

For the hindlimbs (metatarsal region), the corresponding measurement points were:

- metatarsophalangeal (MTP) joint—fetlock;
- metatarsal proximal interphalangeal (PIP) joint—pastern;
- metatarsal distal interphalangeal (DIP) joint—coffin.

At each measurement point, the temperature value was determined from a defined region of interest (ROI). For each limb, the mean limb temperature (MLT) was calculated as the arithmetic average of the three measurement points.

#### 2.3.3. Teat Thermography

Teat surface temperature was recorded for each individual udder quarter: left front (LF), right front (RF), left rear (LR), and right rear (RR). Thermographic images were obtained from a slightly caudo-lateral view, with all four teats visible in one frame whenever feasible. Temperature for each teat was determined from a predefined ROI positioned in the middle region of the teat.

### 2.4. California Mastitis Test

Before milking, the California Mastitis Test (CMT) was performed as a rapid cow-side screening method for the detection of subclinical mastitis. Foremilk samples from each udder quarter were collected separately into the four wells of a CMT paddle after discarding the first streams of milk. An equal volume of commercially available CMT reagent containing alkyl aryl sulphonate detergent was added to each well. The paddle was rotated gently in a circular motion for approximately 10 s to allow mixing of milk and reagent.

The reaction was evaluated visually based on gel formation and viscosity changes caused by the interaction between the reagent and somatic cell DNA. CMT scores were interpreted using the standard scoring system: 0 = negative (no reaction), trace = slight precipitate, 1 = weak positive, 2 = distinct gel formation, and 3 = strong gel formation with marked viscosity increase.

For statistical analysis, CMT results were dichotomized into negative (score 0) and positive (score ≥ 1) categories to distinguish healthy and mastitis-affected udder quarters.

### 2.5. Milk Composition Analysis

At the time of sampling, all cows were clinically free of visible udder inflammation, including swelling, hardness, pain on palpation, redness, or visible clots in milk. During milking, milk samples (*n* = 97) were collected from each cow for determination of fat (%), protein (%), lactose (%), somatic cell count (SCC; ×10^3^ cells/mL), and urea (mg/dL). Samples were transported within 24 h at ≤6 °C to Pieno tyrimai, Joint Stock Company (JSC, Lithuanian: UAB), Kaunas, Lithuania, an ISO/IEC 17025:2018-accredited testing laboratory (certificate No. LA.01.106) [19].

Somatic cell count was determined by flow cytometry using a Somascope cell counter (Foss, Hillerød, Denmark) in accordance with LST EN ISO 13366-1:2008+AC:2009 [20]. Lactose, fat, urea, and milk protein were measured by infrared spectrophotometry using a LactoScope FTIR analyzer (Delta Instruments, Drachten, The Netherlands).

### 2.6. Orthopedic Clinical Examination

After completion of thermographic imaging, the California Mastitis Test (CMT), and milking procedures, all four limbs of each cow underwent systematic orthopedic examination to identify and classify claw pathologies. Orthopedic evaluation was performed approximately 30–60 min after milking. Initially, locomotion was assessed by a trained veterinarian using the five-point locomotion scoring system described by Sprecher et al. [8], based on back posture and gait. Cows were observed both standing and walking on a flat, firm surface. Animals with locomotion scores ≥ 3 were classified as clinically lame.

Following locomotion assessment, detailed claw inspection was performed in all 97 cows using a hydraulic hoof-trimming chute restraint system. Prior to claw examination, the sole surface was washed to facilitate lesion identification. Lesions were classified according to ICAR Claw Health Atlas terminology. Assessed lesion types included digital dermatitis, sole ulcer, white line disease, heel horn erosion, interdigital hyperplasia, sole hemorrhage, and overgrown claws.

Based on the combined orthopedic and claw examination findings, each limb was classified as pathological or non-pathological. Pathological classification was based on orthopedic and claw examination findings rather than locomotion score alone. Mild claw lesions and early-stage pathological changes were also included in the pathological category. This classification approach was intended to minimize potential misclassification of subclinical claw lesions during thermographic evaluation. Forelimbs and hindlimbs were subsequently grouped according to pathological status for statistical analyses.

### 2.7. Statistical Analyses

All statistical analyses and data visualization were performed using RStudio with R version 4.5.2 (R Foundation for Statistical Computing, Vienna, Austria; https://www.r-project.org/, accessed on 30 April 2026). The following R packages were used for statistical analysis and visualization: tidyverse (v2.0.0), ggplot2 (v4.0.3), rstatix (v0.7.3), effectsize (v1.0.2), pROC (v1.19.0.1), lme4 (v2.0-1), psych (v2.6.5), ppcor (v1.1), corrplot (v0.95), qgraph (v1.9.8), ggdist (v3.3.3), and gghalves (v0.1.4). Statistical significance was set at *p* < 0.05.

Data normality was assessed using the Shapiro–Wilk test. Descriptive statistics are presented as median and interquartile range (IQR).

Differences in limb temperature parameters, including coffin (DIP—distal interphalangeal joint), pastern (PIP—proximal interphalangeal joint), fetlock (MCP—metacarpophalangeal joint/MTP—metatarsophalangeal joint), and mean limb temperature (MLT), between groups were evaluated using the Mann–Whitney U test when two independent groups were compared (e.g., limbs with and without pathology or forelimbs versus hindlimbs). When comparisons involved more than two groups (e.g., different limb numbers), the Kruskal–Wallis test was applied. For lesion-specific thermographic analyses, pathological limbs were further classified into infectious lesions (Digital Dermatitis and Interdigital Dermatitis) and claw horn/mechanical lesions (Interdigital Hyperplasia, Double Sole, Sole Ulcer, Sole Haemorrhage, White Line Disease, Heel Horn Erosion, and Abnormal Claw Shape). Thermographic measurements were compared among healthy limbs, infectious lesions, and claw horn/mechanical lesions using the Kruskal–Wallis test. When statistically significant differences were identified, Dunn’s post hoc test with Bonferroni correction was applied for pairwise comparisons.

Effect sizes were calculated to quantify the magnitude of group differences. For Mann–Whitney comparisons, the effect size *r* was calculated as:

(1) $$r=\frac{Z}{\sqrt{N}};$$

where *Z* is the standardized test statistic and *N* is the total number of observations. Effect sizes were interpreted as small (≥0.1), medium (≥0.3), and large (≥0.5). For comparisons involving more than two groups, epsilon-squared (ε^2^) was calculated as a measure of effect size for the Kruskal–Wallis test and interpreted according to commonly used thresholds, where ε^2^ > 0.14 indicates a large effect.

For udder thermography, teat surface temperatures of individual udder quarters (left front (LF), right front (RF), left rear (LR), and right rear (RR)) were analyzed in relation to California Mastitis Test (CMT) results. CMT results were classified as negative (0) or positive (1). Differences between CMT-positive and CMT-negative quarters were assessed using the Mann–Whitney U test, and effect sizes were calculated.

Associations between thermographic measurements and milk composition parameters were evaluated using Spearman’s rank correlation. Partial correlation analysis was performed to assess the relationship between limb pathology (forelimb and hindlimb) and teat surface temperature while controlling for milk composition variables.

A logistic regression model was used to evaluate the association between teat surface temperature and mastitis status. In addition, a mixed-effects logistic regression model was applied to account for the hierarchical structure of the data, with individual quarters nested within cows. In this model, mastitis status (CMT result) was included as the dependent variable, teat surface temperature, parity, and days in milk (DIM) as fixed effects, and cow identity as a random effect.

Receiver operating characteristic (ROC) analysis was performed to evaluate the diagnostic performance of teat surface temperature for identifying CMT-positive udder quarters. The area under the ROC curve (AUC) was calculated as a measure of diagnostic accuracy, and the optimal temperature threshold was determined based on Youden’s index.

Based on ROC results and the distribution of teat surface temperatures in CMT-negative quarters, thermographic diagnostic zones were established to facilitate clinical interpretation. Temperature ranges corresponding to low, moderate, and high probabilities of mastitis were established. No animals were excluded from the study after enrollment. Only thermographic images affected by artefacts were excluded from image analysis.

## 3. Results

### 3.1. Comparison of Surface Temperature Between Individual Limbs of Cows

The Shapiro–Wilk test indicated that all analyzed continuous variables, including coffin (DIP), pastern (PIP), fetlock (MCP/MTP), and mean limb temperature (MLT), significantly deviated from normal distribution (all *p* < 0.05). Therefore, non-parametric statistical methods were applied. Specifically, differences between two independent groups were evaluated using the Mann–Whitney U test, whereas comparisons among more than two groups were performed using the Kruskal–Wallis test followed by Bonferroni-adjusted post hoc comparisons when appropriate. Significant differences between limb numbers were observed for all evaluated thermographic indicators (Table 1).

The Kruskal–Wallis test revealed statistically significant differences among limb numbers for all thermographic measurements. For the coffin joint, a significant effect of limb number was observed (H = 124.43, *p* < 0.001, ε^2^ = 0.318), with post hoc Bonferroni comparisons indicating differences between several limb pairs, as reflected by different superscript letters in Table 1.

Similarly, significant differences were found for pastern (H = 122.42, *p* < 0.001, ε^2^ = 0.313), fetlock (H = 106.65, *p* < 0.001, ε^2^ = 0.271), and mean limb temperature (MLT) (H = 124.97, *p* < 0.001, ε^2^ = 0.319).

Overall, large effect sizes were observed for all thermographic parameters (ε^2^ = 0.271–0.319).

Because no statistically significant differences were detected between the left and right forelimbs or hind limbs for any of the temperature measurement points (Table 1), further analyses were conducted using pooled data from the left and right sides of the forelimbs and hindlimbs.

### 3.2. Comparison Limb Temperature Indicators Between Forelimbs and Hindlimbs

Because no statistically significant bilateral differences were detected between the left and right forelimbs or hindlimbs (Table 1), subsequent analyses were performed using pooled forelimb and hindlimb data (Table 2). Since forelimbs and hindlimbs represented two independent groups, statistical differences were assessed using the Mann–Whitney U test (Table 2).

Statistically significant differences between forelimbs and hindlimbs were observed for all evaluated thermographic indicators, with higher median temperatures consistently recorded in the hindlimbs. For the coffin joint, hindlimb temperatures were significantly higher than those of forelimbs (U = 6406.5, *p* < 0.001, *r* = 0.567). Comparable differences were identified for the pastern (U = 6502.5, *p* < 0.001, *r* = 0.563), fetlock (U = 7334.0, *p* < 0.001, *r* = 0.524), and MLT (U = 6373.5, *p* < 0.001, *r* = 0.569). Effect sizes ranged from 0.524 to 0.569, indicating moderate-to-large effects.

Subsequently, forelimbs and hindlimbs were classified according to the presence or absence of pathology based on thermographic measurements and clinical examination findings, and further analyses were performed using this classification.

### 3.3. Comparison Between Forelimbs with and Without Pathology

Statistically significant differences were observed for all evaluated thermographic indicators. For all evaluated thermographic indicators, median temperatures were significantly higher in pathological forelimbs than in non-pathological forelimbs (Table 3).

Median temperatures were significantly higher in pathological forelimbs than in non-pathological forelimbs across all evaluated thermographic indicators. For the coffin joint, median temperatures were 27.55 °C and 18.00 °C in pathological and non-pathological forelimbs, respectively (U = 10.5, *p* < 0.001, *r* = 0.669). Comparable differences were identified for the pastern (median 26.55 vs. 18.50; U = 61.5, *p* < 0.001, *r* = 0.657), fetlock (median 26.70 vs. 18.65; U = 81.5, *p* < 0.001, *r* = 0.652), and MLT (median 27.12 vs. 18.47; U = 6.5, *p* < 0.001, *r* = 0.670). Effect sizes ranged from 0.652 to 0.670, indicating large effects across all evaluated parameters.

### 3.4. Comparison Between Hindlimbs with and Without Pathology

Statistically significant differences were observed for all evaluated thermographic indicators. For all evaluated thermographic indicators, median temperatures were significantly higher in pathological hindlimbs than in non-pathological hindlimbs (Table 4).

Median temperatures were significantly higher in pathological hindlimbs than in non-pathological hindlimbs across all evaluated thermographic indicators. For the coffin joint, median temperatures were 30.30 °C and 19.10 °C in pathological and non-pathological hindlimbs, respectively (U = 0.0, *p* < 0.001, *r* = 0.797). Comparable differences were identified for the pastern (median 29.10 vs. 19.80; U = 12.0, *p* < 0.001, *r* = 0.795), fetlock (median 28.20 vs. 19.50; U = 1.0, *p* < 0.001, *r* = 0.797), and MLT (median 29.00 vs. 19.47; U = 0.0, *p* < 0.001, *r* = 0.797). Effect sizes ranged from 0.795 to 0.797, indicating very large effects across all evaluated parameters.

Both anatomical location and pathological status significantly influenced joint temperature measurements. Consistent differences were observed across all evaluated anatomical sites, including the coffin (DIP), pastern (PIP), and fetlock (MCP/MTP) joints, as well as mean limb temperature (MLT).

### 3.5. Comparison of Thermographic Measurements Among Healthy Forelimbs, Infectious Lesions, and Claw Horn Lesions

Among the forelimbs, 158 limbs were classified as healthy, 30 as having claw horn/mechanical lesions, and 6 as having infectious lesions. Infectious lesions included Digital Dermatitis (DD) and Interdigital Dermatitis (ID), whereas claw horn/mechanical lesions comprised Double Sole (DS), Sole Ulcer (SU), Heel Horn Erosion (HHE), and Abnormal Claw Shape (AC). Significant differences were observed among groups for all evaluated thermographic indicators (Table 5). Healthy forelimbs consistently exhibited lower temperatures than limbs affected by either infectious or claw horn/mechanical lesions.

Median temperatures were significantly higher in both infectious and claw horn/mec7hanical lesions than in healthy forelimbs across all evaluated anatomical locations (Table 5). For coffin, median temperatures were 18.00, 27.55, and 27.65 °C in healthy, claw horn/mechanical, and infectious lesions, respectively (H = 86.91, *p* < 0.001, ε^2^ = 0.445). Similar patterns were observed for pastern, fetlock, and MLT, with large effect sizes across all thermographic indicators (ε^2^ = 0.423–0.446). Post hoc comparisons demonstrated significantly higher temperatures in both pathological groups than in healthy limbs (*p* < 0.001), whereas no significant differences were detected between infectious and claw horn/mechanical lesions (*p* > 0.05).

### 3.6. Comparison of Thermographic Measurements Among Healthy Hindlimbs, Infectious Lesions, and Claw Horn Lesions

Among the hindlimbs, 59 limbs were classified as healthy, 104 as having claw horn/mechanical lesions, and 29 as having infectious lesions. Infectious lesions included Digital Dermatitis (DD) and Interdigital Dermatitis (ID), whereas claw horn/mechanical lesions comprised Interdigital Hyperplasia (IH), Double Sole (DS), Sole Ulcer (SU), Sole Hemorrhage (SH), White Line Disease (WLD), Heel Horn Erosion (HHE), and Abnormal Claw Shape (AC). Significant differences were observed among groups for all evaluated thermographic indicators (Table 6). Healthy hindlimbs consistently exhibited lower temperatures than limbs affected by either infectious or claw horn/mechanical lesions.

Median temperatures differed significantly among healthy hindlimbs, claw horn/mechanical lesions, and infectious lesions for all evaluated thermographic indicators (Table 6). For the coffin joint, median temperatures were 19.10 °C, 30.05 °C, and 30.80 °C, respectively (H = 123.73, *p* < 0.001, ε^2^ = 0.644). Similar patterns were observed for the pastern, fetlock, and mean limb temperature (MLT), with all comparisons remaining statistically significant (H = 124.46–126.33, *p* < 0.001) and associated with large effect sizes (ε^2^ = 0.644–0.658).

Post hoc comparisons revealed significantly higher temperatures in both infectious and claw horn/mechanical lesions than in healthy hindlimbs across all evaluated thermographic indicators (*p* < 0.001). No significant difference was observed between infectious and claw horn/mechanical lesions for the coffin joint temperature (*p* = 0.059), whereas infectious lesions exhibited significantly higher temperatures than claw horn/mechanical lesions for PIP, MTP, and MLT (*p* < 0.05).

### 3.7. Relationship Between Teat Surface Temperature and CMT Results

**Figure 2:**
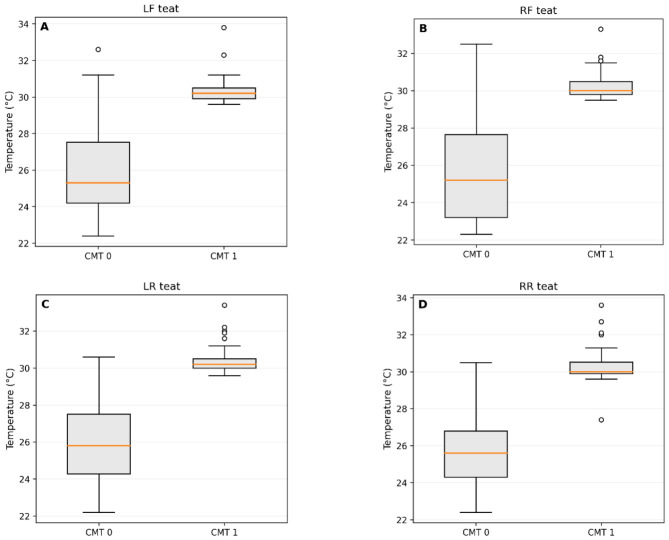
Differences in teat surface temperature between CMT-negative and CMT-positive udder quarters. Panels (**A**–**D**) show boxplots of teat surface temperature for left front (LF), right front (RF), left rear (LR), and right rear (RR) quarters according to California Mastitis Test (CMT) results. The numbers of analyzed quarters were as follows: CMT-negative quarters—LF (*n* = 64), RF (*n* = 68), LR (*n* = 68), and RR (*n* = 65); CMT-positive quarters—LF (*n* = 33), RF (*n* = 29), LR (*n* = 29), and RR (*n* = 32). The orange horizontal line within each box represents the median, the boxes indicate the interquartile range (IQR), the whiskers extend to 1.5 × IQR, and the open circles represent outliers.

Statistically significant differences in teat surface temperature were observed across all udder quarters. In each case, CMT-positive quarters exhibited higher teat surface temperatures compared with CMT-negative quarters (Figure 2).

Thermographic measurements of teat surface temperature were significantly associated with mastitis status determined by the CMT. Across all udder quarters, CMT-positive quarters demonstrated markedly higher temperatures compared with CMT-negative quarters.

Median temperatures ranged from 25.2 to 25.8 °C in CMT-negative quarters and from 30.0 to 30.2 °C in CMT-positive quarters. The Mann–Whitney U test confirmed statistically significant differences in all four quarters (*p* < 0.001), with large effect sizes observed across all quarters (*r* = 0.688–0.750).

### 3.8. Diagnostic Performance of Teat Surface Temperature for Mastitis Detection

**Figure 3:**
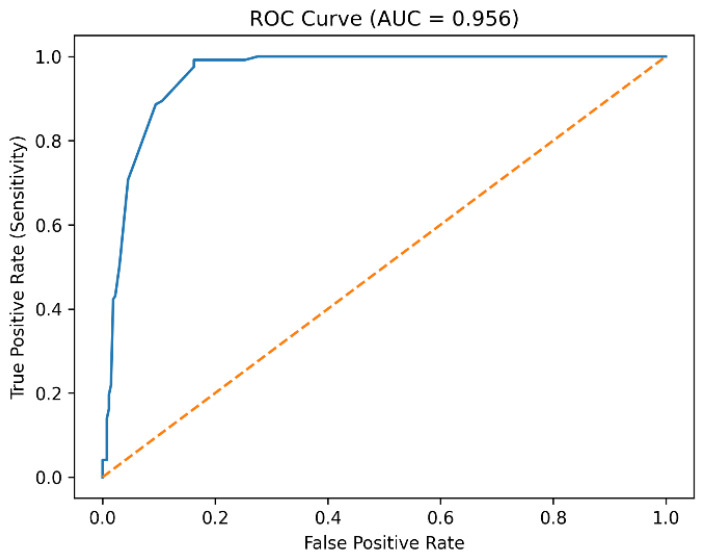
Receiver operating characteristic (ROC) curve evaluating teat surface temperature for detection of CMT-positive udder quarters. ROC curve evaluating the diagnostic ability of teat surface temperature for detecting CMT-positive quarters. The area under the curve (AUC) was 0.956, indicating excellent diagnostic accuracy, and the optimal temperature threshold identified using the Youden index was approximately 29.5 °C. The orange dotted diagonal line represents the line of no discrimination (random classification; AUC = 0.5), which serves as the reference for evaluating the diagnostic performance of the ROC curve.

In this analysis, data from all udder quarters (LF, RF, LR, RR) were combined, with each observation representing a single quarter with its corresponding temperature and CMT result. Receiver operating characteristic (ROC) analysis was then performed to evaluate the diagnostic performance of teat surface temperature for identifying CMT-positive udder quarters (Figure 3).

The analysis demonstrated excellent diagnostic accuracy, with an area under the ROC curve (AUC) of 0.956 (95% CI: 0.930–0.982). The optimal temperature threshold for detecting CMT-positive quarters was 29.5 °C, as determined using the Youden index. At this cut-off value, teat surface temperature identified mastitis-positive quarters with a sensitivity of 0.992 and a specificity of 0.838.

### 3.9. Relationship Between Teat Surface Temperature and Milk Composition

Spearman correlation analysis was performed to evaluate the relationship between mean teat surface temperature and milk composition parameters (Table 7).

No statistically significant correlations were observed between teat temperature and milk fat, lactose, or somatic cell count (SCC) (*p* > 0.05). However, weak but statistically significant negative correlations were found between teat surface temperature and both milk protein (r = −0.219, *p* = 0.031) and milk urea concentration (r = −0.234, *p* = 0.021).

Overall, these results indicate that thermographic measurements of teat surface temperature show limited association with general milk composition parameters, although modest relationships with protein and urea concentrations were observed.

### 3.10. Relationship Between Teat Surface Temperature and Somatic Cell Count

**Figure 4:**
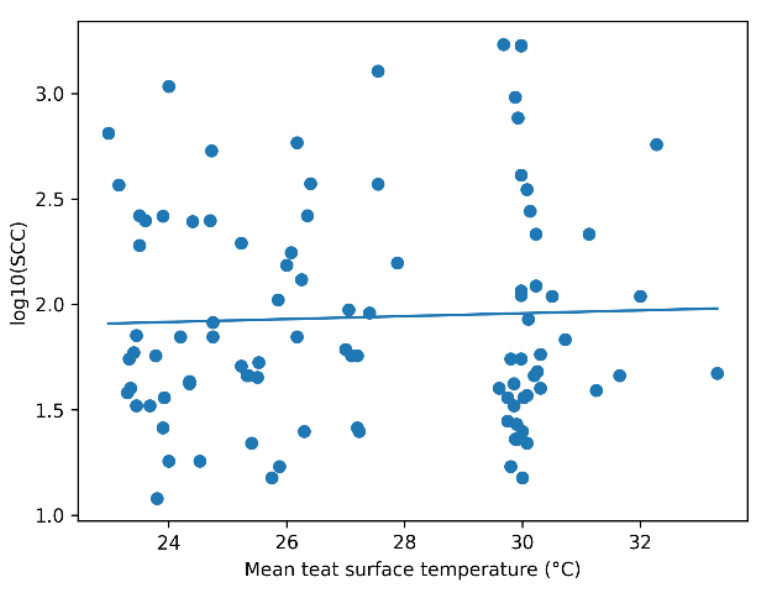
Linear regression between mean teat surface temperature and logarithmically transformed somatic cell count (log_10_SCC).

A linear regression analysis (Figure 4) was performed to evaluate the relationship between mean teat surface temperature and the logarithmically transformed somatic cell count (log_10_SCC).

A linear regression analysis revealed no significant association between mean teat surface temperature and log_10_SCC (r = 0.037, *p* = 0.719), with a negligible regression slope (β = 0.0069). These results indicate that teat temperature does not meaningfully reflect variations in composite milk somatic cell count, despite its strong association with CMT status.

### 3.11. Mean Teat Surface Temperature According to SCC Group

**Figure 5:**
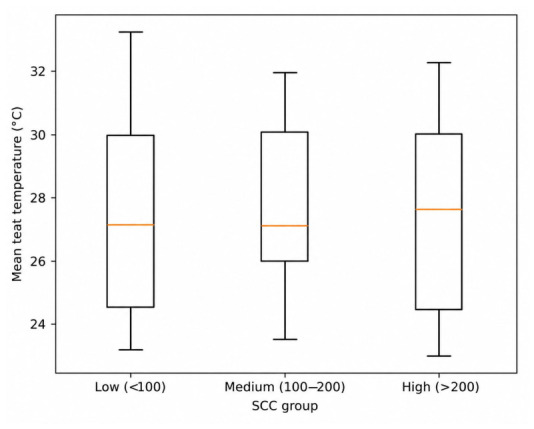
Mean teat surface temperature according to somatic cell count (SCC) group. SCC groups were defined as low (<100 × 10^3^ cells/mL; *n* = 60), medium (100–200 × 10^3^ cells/mL; *n* = 12), and high (>200 × 10^3^ cells/mL; *n* = 25). The orange horizontal line within each box represents the median, the boxes indicate the interquartile range (IQR), and the whiskers extend to 1.5 × IQR.

To further investigate the relationship between teat surface temperature and milk quality, cows were classified into three groups based on somatic cell count (SCC): low (<100 × 10^3^ cells/mL), medium (100–200 × 10^3^ cells/mL), and high (>200 × 10^3^ cells/mL) (Figure 5).

Median mean teat surface temperatures were 27.08 °C (IQR 24.48–29.94) in the low SCC group, 27.06 °C (IQR 25.96–30.04) in the medium SCC group, and 27.55 °C (IQR 24.40–29.98) in the high SCC group. The Kruskal–Wallis test indicated no statistically significant differences in mean teat temperature among SCC groups (H = 0.86, *p* = 0.652).

These findings suggest that teat surface temperature measured by thermography is not strongly associated with composite milk somatic cell count, although clear associations were previously observed with quarter-level CMT results. This difference may reflect the fact that SCC was determined from composite milk samples at the cow level, whereas both thermographic measurements and CMT assessments were performed at the quarter level.

### 3.12. Mean Teat Surface Temperature According to the Number of CMT-Positive Quarters

**Figure 6:**
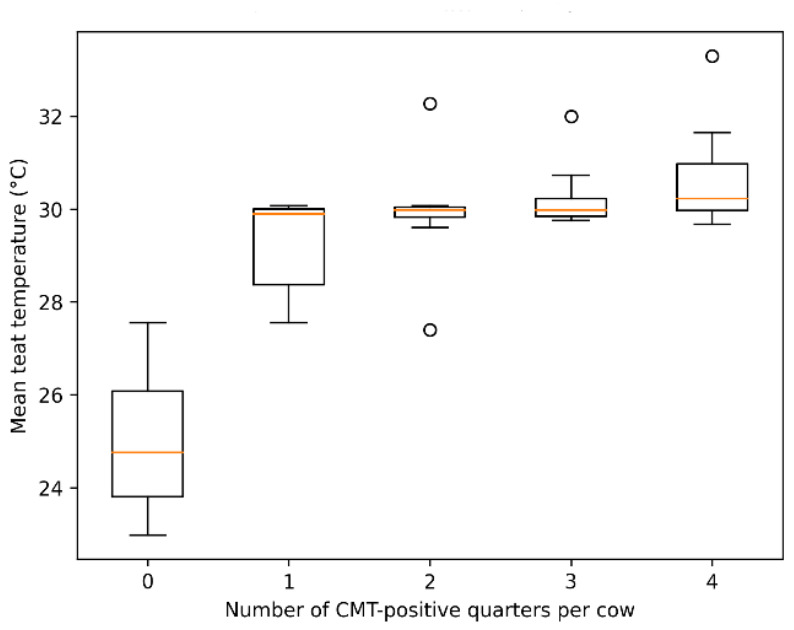
Mean teat surface temperature according to number of CMT-positive udder quarters per cow. Boxplots show mean teat surface temperature in cows with 0 (*n* = 53), 1 (*n* = 6), 2 (*n* = 11), 3 (*n* = 13), and 4 (*n* = 14) CMT-positive quarters. The orange horizontal line within each box represents the median, the boxes indicate the interquartile range (IQR), the whiskers extend to 1.5 × IQR, and the open circles represent outliers.

To further investigate the relationship between thermographic measurements and mastitis severity, cows were classified according to the number of CMT-positive quarters (0–4) (Figure 6).

Mean teat surface temperature increased markedly with the number of CMT-positive quarters. The median values of mean teat surface temperature ranged from 24.75 °C (IQR 23.80–26.08) in cows without CMT-positive quarters to 30.23 °C (IQR 29.97–30.97) in cows with four CMT-positive quarters.

The Kruskal–Wallis test demonstrated statistically significant differences between groups (H = 72.72, *p* < 0.001), and Spearman correlation analysis confirmed a very strong positive association between mean teat surface temperature and the number of CMT-positive quarters (r = 0.855, *p* < 0.001).

These findings indicate that teat surface temperature measured by infrared thermography increases substantially with the extent of mastitis involvement within the udder.

### 3.13. Mixed-Effects Model for Mastitis Prediction

A mixed-effects logistic regression model was used to evaluate the association between teat surface temperature and mastitis status while accounting for clustering of quarters within cows and adjusting for parity and days in milk (DIM) (Table 8).

The model demonstrated a significant positive association between teat surface temperature and the probability of a CMT-positive quarter (β = 1.67, SE = 0.43, z = 3.88, *p* < 0.001). The corresponding odds ratio indicated that each 1 °C increase in teat surface temperature increased the odds of a quarter being CMT-positive by approximately 5.3-fold (OR = 5.30; 95% CI: 2.28–12.31).

In contrast, neither parity nor DIM showed statistically significant associations with CMT status (*p* > 0.05).

Overall, these results indicate that teat surface temperature remained a strong independent predictor of mastitis status even after adjustment for parity, DIM, and the hierarchical structure of the data.

### 3.14. Thermographic Diagnostic Zones for Mastitis Detection

**Figure 7:**
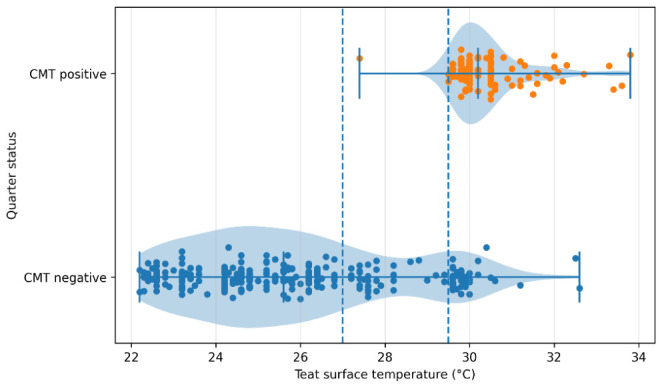
Raincloud plot showing the distribution of teat surface temperatures in CMT-negative and CMT-positive udder quarters. Density distributions are presented as violin plots, while individual observations are displayed as horizontally jittered points. Blue denotes CMT-negative quarters, whereas orange denotes CMT-positive quarters. The vertical blue dashed lines indicate the proposed thermographic diagnostic thresholds at 27 °C and 29.5 °C.

Based on ROC analysis and the distribution of teat temperatures (Figure 7) in CMT-negative quarters, three thermographic diagnostic zones were defined (Table 9).

Temperatures below 27 °C were predominantly associated with healthy udder tissue and a low probability of mastitis. A transition zone between 27 °C and 29.5 °C was identified, in which the probability of mastitis increased and additional diagnostic testing was recommended. Temperatures ≥ 29.5 °C, corresponding to the optimal ROC-derived threshold, were consistently associated with CMT-positive quarters and indicated a high probability of mastitis.

Overall, these thermographic zones provide a practical framework for the clinical interpretation of infrared thermography in mastitis screening.

### 3.15. Correlation Between Forelimb and Hindlimb Pathology, Teat Temperature, and Milk Composition

**Figure 8:**
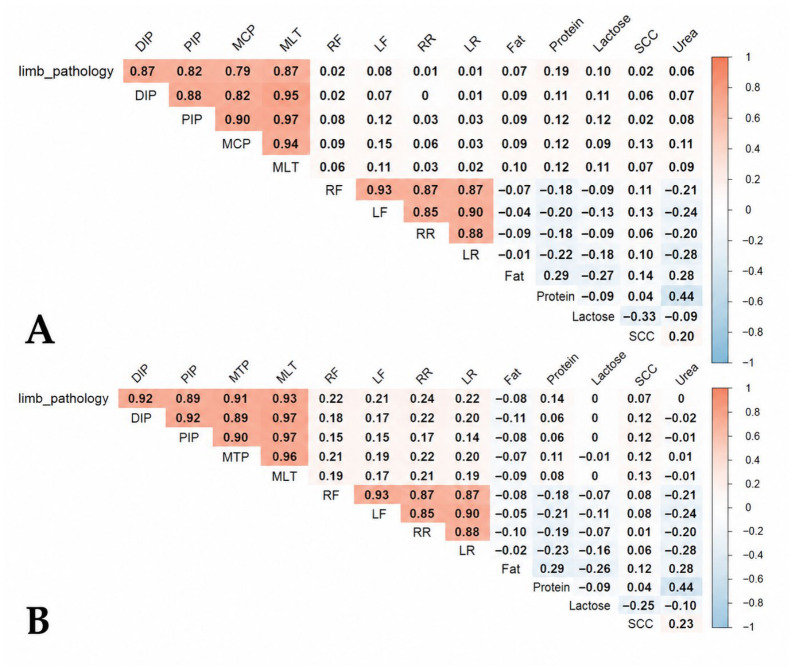
Correlation analysis of limb pathology, thermographic measurements, and milk quality indicators. Spearman correlation matrices showing relationships between limb pathology, thermographic measurements, teat surface temperatures, and milk quality indicators based on data from 97 cows. Panel (**A**): forelimb pathology; Panel (**B**): hindlimb pathology.

Spearman correlation analysis was conducted to investigate relationships between forelimb (Figure 8A) and hindlimb (Figure 8B) pathology, teat surface temperatures, and milk composition parameters.

No statistically significant correlations were observed between forelimb pathology and teat surface temperatures or milk composition parameters (r ≤ 0.19, *p* > 0.05). In contrast, weak but statistically significant positive correlations were identified between hindlimb pathology and teat surface temperatures across all udder quarters (r = 0.21–0.24, *p* < 0.01).

Strong correlations were observed among teat surface temperatures across different udder quarters (r = 0.85–0.93). Weak negative correlations were identified between teat surface temperature and certain milk parameters, particularly protein (r ≈ −0.18 to −0.23) and urea (r ≈ −0.20 to −0.28). Among milk parameters, protein and urea demonstrated a moderate positive correlation (r = 0.44), while lactose showed weak negative correlations with SCC (r = −0.33 in Figure 8A and r = −0.25 in Figure 8B).

Overall, forelimb pathology was not significantly associated with teat thermography or milk composition parameters, whereas hindlimb pathology demonstrated weak positive associations with teat surface temperatures.

### 3.16. Partial Correlation Analysis for Forelimb and Hindlimb Pathology

Partial correlation analysis was performed to evaluate the relationships between limb pathology and teat surface temperature while controlling for milk composition parameters (fat, protein, lactose, SCC, and urea) (Table 10).

No statistically significant associations were observed between forelimb pathology and teat surface temperatures in any udder quarter (r = 0.05–0.13, *p* > 0.05). In contrast, significant positive partial correlations were identified between hindlimb pathology and teat surface temperatures across all udder quarters (r = 0.23–0.26, *p* ≤ 0.001), with the highest coefficients observed for the rear quarters (RR and LR).

## 4. Discussion

### 4.1. Limb Surface Temperature Patterns

Hindlimbs exhibited consistently elevated surface temperatures compared with forelimbs across all anatomical sites (coffin: 28.80 vs. 18.55 °C; MLT: 27.37 vs. 18.96 °C; *p* < 0.001, *r* = 0.524–0.569). This fore–hind thermal gradient has been attributed to greater weight-bearing, increased metabolic activity, and enhanced vascular perfusion of the hindquarters in dairy cattle [1,2]. Gregić et al. [4] confirmed significant temperature differences between front and rear legs at both the coronary band and the skin in Holstein cows, further demonstrating that leg position is a key determinant of baseline thermographic values. No left–right differences were detected in healthy limbs, confirming bilateral thermal symmetry. This symmetry enables use of the contralateral limb as an internal reference, controlling for inter-individual and environmental variation [1,4]. Beyond these physiological explanations, several environmental and positional factors are likely to accentuate the observed forehind temperature gradient [1,2,4,12]. Hindlimbs are more frequently in close contact with manure-contaminated and moist flooring in free-stall barns, which may alter local thermal conductivity and convective heat loss [1,17,18]. During imaging in the milking parlour, cows also tended to stand with the hindlimbs positioned closer to the camera and more directly exposed to the field of view, whereas the forelimbs were partially shielded by the thorax and shoulder region, which may have increased the apparent temperature contrast [1,2,4,10]. In addition, differences in stance, weight distribution, and hoof–floor contact area between fore- and hindlimbs can influence local perfusion and heat dissipation, further contributing to the marked baseline temperature difference we observed [1,2,21].

Pathological limbs showed markedly elevated temperatures (hindlimb coffin: 30.30 vs. 19.10 °C, r around 0.80; forelimb coffin: 27.55 vs. 18.00 °C, *r* = 0.652–0.670), consistent with inflammation-induced vasodilation and hyperemia [1,22]. These values exceed some diagnostic thresholds reported in recent studies: Werema et al. [7] identified a cut-off of 38.0 °C for foot skin temperature (sensitivity 73.2%, specificity 86.0%) for lameness detection, while Coe and Blackie [23] demonstrated that both low-cost and standard commercial IRT devices could discriminate lame from sound cows based on maximum adjusted temperature and hind feet temperature differences. Although the optimal foot skin temperature cut-off of 38.0 °C reported by Werema et al. [7] appears numerically higher than the temperatures observed in the present study, this discrepancy is expected given substantial methodological and biological differences between studies. Werema et al. [7] measured mean foot skin temperature on the plantar aspect of the hind feet under different ambient conditions and evaluated lameness defined by locomotion score, whereas our measurements were obtained on the dorsal coffin region in a milking parlour at approximately 15 °C and primarily aimed at characterizing thermographic patterns rather than establishing a universal lameness threshold. Absolute IRT values are strongly affected by measurement site, hair coverage, ambient temperature and airflow, and local tissue perfusion, so diagnostic cut-offs are inherently context- and protocol-specific and should not be directly compared across studies or anatomical regions. The larger effect sizes observed for hindlimb pathology likely reflect the higher prevalence of claw disorders in the hindlimbs and the lower forelimb baseline temperature, which reduces absolute thermal contrast [4,21]. Bobić et al. [1] reviewed recent evidence confirming that disease presence, leg position, measurement view, and ambient temperature all significantly influence recorded foot temperatures, underscoring the need for standardized protocols. A recent pilot study by Ferrara et al. [22] demonstrated that IRT-detected foot temperatures in dairy cows with lesions were significantly elevated compared with healthy controls (*p* < 0.01), with temperature normalization observed following treatment, confirming the utility of IRT for both diagnosis and post-treatment monitoring. Additional lesion-specific analyses demonstrated that both infectious and claw horn/mechanical lesions were associated with significantly elevated thermographic values compared with healthy limbs. Interestingly, no significant differences were observed between infectious and claw horn/mechanical lesions in the forelimbs, whereas infectious lesions exhibited significantly higher temperatures than claw horn/mechanical lesions in the hindlimbs. This finding is biologically plausible because infectious claw disorders, particularly Digital Dermatitis and Interdigital Dermatitis, are characterized by active inflammatory processes and increased local vascular perfusion, which may generate stronger thermal responses than chronic claw horn lesions. The effect was most apparent in the hindlimbs, where infectious claw disorders are more prevalent and typically more severe in dairy cattle. Álvarez et al. [24] further reported that coronary band temperatures were significantly elevated in cows with locomotion scores ≥ 3, and that parity influenced baseline hoof temperature, indicating the need for stratified reference ranges.

### 4.2. Infrared Thermography for Mastitis Detection

CMT-positive quarters exhibited teat surface temperatures of 30.0–30.2 °C compared with 25.2–25.8 °C in CMT-negative quarters (*p* < 0.001, *r* = 0.688–0.750), corresponding to a differential of approximately 4.5–5.0 °C. Gelasakis et al. [5] reviewed the use of IRT for mastitis detection in dairy ruminants and reported temperature differentials ranging from 1.0 to 9.6 °C depending on infection severity, measurement site, and environmental conditions. Gayathri et al. [10] reported significant differences in udder and teat skin surface temperatures between healthy and mastitic quarters across all seasons, with strong correlations with CMT scores and log_10_SCC (*p* < 0.01), thereby confirming the robustness of the IRT–mastitis relationship under varying environmental conditions. Silva et al. [15] likewise confirmed that IRT detected udder temperature increases in CMT-positive cows in a pasture-based grazing system.

ROC analysis demonstrated excellent discriminatory performance (AUC = 0.956), with an optimal threshold of 29.5 °C (sensitivity 0.992, specificity 0.838). Santana et al. [16] applied machine learning (XGBoost) to IRT-based mastitis diagnosis and achieved an AUC of 0.843 with 93.3% specificity, identifying the coldest point in the region of interest as the most important diagnostic feature. Lisuzzo et al. [6] reviewed on-farm mastitis diagnostics and highlighted IRT as a promising emerging tool, though emphasizing that its application still requires further optimization under field conditions. Based on the present ROC analysis and temperature distributions, three diagnostic zones were defined: <27 °C (healthy quarter), 27–29.5 °C (borderline zone warranting confirmatory testing), and ≥29.5 °C (mastitis likely). This tiered framework may facilitate clinical triage by minimizing both missed diagnoses and unnecessary interventions.

The apparent discrepancy between the strong thermography–CMT association and the absence of a significant relationship with SCC may be explained by differences in sampling scale. Thermographic measurements and CMT results were obtained at the quarter level, whereas SCC values represented composite milk samples from the entire cow. Consequently, localized inflammatory changes within a single udder quarter may have been diluted in the composite milk SCC measurement, reducing the strength of the association with thermographic findings. This interpretation is supported by the present results, which demonstrated strong relationships between teat temperature and quarter-level CMT status but no significant associations with cow-level SCC.

The mixed-effects logistic regression model demonstrated that each 1 °C increase in teat surface temperature was associated with a 5.3-fold increase in the odds of CMT-positivity (OR = 5.30, *p* < 0.001, 95% CI: 2.28–12.31), while appropriately accounting for quarter-within-cow clustering. A strong dose–response relationship was observed between mean teat surface temperature and the number of CMT-positive quarters (*r* = 0.855, *p* < 0.001), with temperatures increasing from 24.75 °C in cows with no positive quarters to 30.23 °C in cows with four positive quarters. This gradient suggests that IRT reflects the overall inflammatory burden of the udder and may therefore have value for cow-level screening. Guedes et al. [3] reviewed precision livestock farming technologies for lameness and mastitis and concluded that integrating IRT with automated milking system data represents a viable approach for real-time udder health monitoring.

### 4.3. Teat Surface Temperature and Milk Composition

No significant correlations were found between teat surface temperature and milk fat, lactose, or composite milk SCC (*p* > 0.05). The absence of an association between teat temperature and SCC (r = 0.037, *p* = 0.719) is consistent with the fundamental mismatch between quarter-level thermographic measurements and composite milk SCC, which aggregates and dilutes quarter-specific inflammatory signals [5,9]. Peckler and Adcock [9] similarly reported that udder skin temperature had the lowest AUC (0.56) for detecting subclinical mastitis in meat-type ewes and noted that ambient temperature exerted greater influence on IRT readings than infection status when quarter-level matching was not performed. These findings underscore that meaningful IRT validation requires quarter-level SCC data.

Weak but significant negative correlations were observed with milk protein (r = −0.219, *p* = 0.031) and urea (r = −0.234, *p* = 0.021), possibly reflecting impaired alveolar epithelial function and altered nitrogen metabolism associated with subclinical inflammation [5,14]. However, given their modest magnitude and bulk-level measurement, these associations should be interpreted with caution.

### 4.4. Association Between Limb Pathology and Udder Health

Forelimb pathology showed no significant associations with teat surface temperatures in either bivariate or partial correlation analyses. In contrast, hindlimb pathology was positively correlated with teat temperatures across all quarters (r = 0.21–0.24, *p* < 0.01), and these associations remained significant after adjustment for milk composition (partial r = 0.23–0.26, *p* ≤ 0.001), indicating a direct biological relationship independent of milk composition-related confounding factors.

The most plausible mechanism involves altered lying behaviour. Cows with hindlimb lameness tend to increase recumbency time to reduce weight-bearing on affected limbs, thereby increasing teat exposure to environmental pathogens [17,25]. Zigo et al. [17] reported that cows with claw disease exhibited significantly elevated mastitis scores compared with non-lame controls, with environmental pathogens predominating, and suggested that this association may be related to increased lying time. Robles et al. [18] further confirmed that dirtier stalls were associated with poorer udder hygiene and higher odds of lameness, supporting a mechanistic link between housing conditions, lying behaviour, and mastitis risk. The anatomical specificity of this relationship to the hindlimbs is consistent with the predominance of hindlimb locomotor disorders in dairy cattle [1,21] and with postural adaptations that may preferentially expose the posterior udder to ground-level contaminants during recumbency. The strong inter-quarter correlations in teat temperature (r = 0.85–0.93) support the interpretation of diffuse environmental pathogen exposure rather than isolated quarter infection.

### 4.5. Clinical Implications and Limitations

These findings support IRT as a dual-purpose screening tool for the simultaneous assessment of limb pathology and mastitis. The three-zone teat temperature classification provides a practical framework for clinical triage, and the association between hindlimb pathology and teat temperature may have important implications for integrated prevention strategies that combine hoof health management with mastitis control [3,17]. Importantly, the diagnostic performance reported in this study reflects accuracy relative to CMT-based classification, which is a widely used cow-side screening test but not a full microbiological gold standard; therefore, the proposed thresholds should be regarded as preliminary until validated against quarter-level SCC and bacteriological culture. Several limitations should be considered when interpreting these results. Because of the cross-sectional design, causal relationships cannot be inferred, and follow-up longitudinal studies that repeatedly assess the same cows over time are needed to clarify the directionality of the observed associations. In addition, data collection was restricted to a single month (October 2025), which did not allow evaluation of potential seasonal variation in limb pathology, udder health, or thermographic measurements, and therefore limits inference about longer-term epidemiological dynamics. Ambient temperature is a well-recognized confounder of IRT measurements [1,4], so future validation work should incorporate systematic adjustment for environmental conditions. In addition, relative humidity and air velocity were not quantitatively measured during image acquisition and therefore could not be incorporated into the statistical analyses. Future studies should evaluate the potential influence of these environmental factors on thermographic measurements under commercial farm conditions. Furthermore, the strict exclusion of thermographic images due to manure contamination or moisture represents a major limitation for the seamless automation of IRT in commercial free-stall barns, where distal limbs are frequently soiled. Future algorithms must incorporate automated image-cleaning or advanced filtering to overcome this operational bottleneck. In addition, neither quarter-level bacteriological culture nor individual quarter SCC measurements were available for validation of mastitis status. Consequently, the diagnostic performance of thermography was evaluated relative to CMT classification, and further validation against microbiological and quarter-level SCC data is warranted.

Furthermore, the use of a single-herd study population may limit the generalizability of the proposed thresholds across breeds, management systems, and climatic conditions. In addition, some subgroup analyses involved relatively limited pathological subgroup sizes, which may have reduced the statistical power to detect weaker associations or smaller effect sizes. In particular, the number of infectious forelimb lesions was relatively small, which may have limited statistical power for detecting differences between infectious and claw horn/mechanical lesion categories in the forelimbs. Moreover, although detailed claw inspection was performed in all cows, the possibility of undetected subclinical or very early-stage claw lesions cannot be completely excluded, which may have contributed to reduced apparent specificity of thermographic classification in some cases. Therefore, the present results should be regarded as most directly applicable to high-yield Holstein–Friesian cows kept in similar free-stall housing and temperate climatic conditions rather than universally generalizable across all dairy production contexts. Future research should prioritize longitudinal multi-herd designs with quarter-level microbiological validation and integration of IRT data into precision livestock farming platforms [3,16].

The structured thermographic and clinical data generated in this study also provide a suitable foundation for AI-based models, and integrating such algorithms with IRT-derived features and precision livestock farming systems may further enhance automated detection and real-time monitoring of limb pathology and mastitis in commercial dairy herds.

## 5. Conclusions

This study demonstrated that infrared thermography may serve as a feasible non-invasive screening tool for the detection of limb pathology and subclinical mastitis in dairy cows. In addition, the findings provide a valuable baseline dataset for the development of machine learning algorithms capable of identifying relationships between limb disorders and udder diseases in lactating dairy cows. Hindlimbs exhibited physiologically elevated surface temperatures compared with forelimbs, whereas bilateral thermal symmetry in healthy animals supports the use of the contralateral limb as a diagnostic reference. Limb pathology was associated with significantly elevated surface temperatures, with very large effect sizes observed in the hindlimbs (r around 0.80). Lesion-specific analyses further demonstrated that both infectious and claw horn/mechanical disorders were associated with elevated thermographic values compared with healthy limbs. In the hindlimbs, infectious lesions exhibited higher temperatures than claw horn/mechanical lesions, indicating that thermographic responses may vary according to lesion type and inflammatory activity. Teat surface temperature provided excellent diagnostic accuracy for identifying CMT-positive udder quarters (AUC = 0.956; optimal threshold 29.5 °C; sensitivity 0.992; specificity 0.838), with each 1 °C increase corresponding to a 5.37-fold increase in the odds of subclinical mastitis (OR = 5.37, *p* < 0.001). A clear dose–response relationship between mean teat surface temperature and the number of CMT-positive quarters per cow (r = 0.855, *p* < 0.001) indicates that thermography reflects the overall udder inflammatory burden. The three-zone thermographic classification (<27 °C: healthy; 27–29.5 °C: monitoring recommended; ≥29.5 °C: mastitis likely) offers a practical on-farm triage framework for mastitis screening, while the lack of significant associations between teat surface temperature and composite milk somatic cell count, fat, or lactose confirms that thermography captures localized quarter-level inflammation not reflected in composite milk indicators. Hindlimb pathology was independently associated with elevated teat surface temperatures (partial r = 0.23–0.26, *p* ≤ 0.001), whereas forelimb pathology showed no such association, suggesting that hindlimb locomotor disorders may increase mastitis risk through altered lying behaviour and increased environmental pathogen exposure. Future studies employing longitudinal multi-herd designs with quarter-level bacteriological validation are warranted to confirm the proposed diagnostic thresholds and to further elucidate the causal relationship between hindlimb pathology and udder health.

## Figures and Tables

**Table 1:** Thermographic limb temperature indicators (°C) according to individual limb location (*n* = 388 limbs).

Thermographic Measurements	FRL	FLL	RRL	RLL	H	*p*-Value	ε^2^
Coffin	18.40 (17.20–20.90) ^a^	18.60 (17.20–20.70) ^a^	29.20 (20.25–30.90) ^b^	28.25 (20.08–30.80) ^b^	124.43	*p* < 0.001	0.318
Pastern	19.10 (17.70–21.50) ^a^	18.80 (17.50–21.20) ^a^	27.60 (20.77–29.52) ^b^	26.70 (21.35–29.50) ^b^	122.42	*p* < 0.001	0.313
Fetlock	19.40 (18.20–22.30) ^a^	19.40 (18.00–22.60) ^a^	27.05 (20.62–28.42) ^b^	27.10 (21.10–28.85) ^b^	106.65	*p* < 0.001	0.271
MLT	18.87 (17.93–20.97) ^a^	19.10 (17.77–21.40) ^a^	28.05 (20.66–29.60) ^b^	26.84 (20.68–29.70) ^b^	124.97	*p* < 0.001	0.319

Data are presented as median (interquartile range, IQR). H denotes the Kruskal–Wallis test statistic, and ε^2^ (epsilon squared) was used to estimate effect size. Different superscript letters within a row indicate significant differences between limb numbers according to Bonferroni-adjusted post hoc comparisons (*p* < 0.05). FRL = front right limb; FLL = front left limb; RRL = rear right limb; RLL = rear left limb; MLT = mean limb temperature.

**Table 2:** Comparison of thermographic temperature indicators (°C) between forelimbs (*n* = 194) and hindlimbs (*n* = 194).

Thermographic Measurements	Forelimbs	Hindlimbs	U	*p*-Value	Effect Size (*r*)
Coffin	18.55 (17.20–20.85)	28.80 (20.08–30.82)	6406.50	*p* < 0.001	0.567
Pastern	19.05 (17.62–21.40)	27.10 (21.08–29.50)	6502.50	*p* < 0.001	0.563
Fetlock	19.40 (18.20–22.48)	27.10 (20.80–28.73)	7334.00	*p* < 0.001	0.524
MLT	18.96 (17.82–21.18)	27.37 (20.66–29.67)	6373.50	*p* < 0.001	0.569

Data are presented as median and interquartile range (IQR). Differences between limb numbers were assessed using the Mann–Whitney U test. Effect size is reported as r.

**Table 3:** Comparison of thermographic limb temperature indicators (°C) between pathological and non-pathological forelimbs.

Thermographic Measurements	No Pathology (*n* = 158)	Pathology (*n* = 36)	U	*p*-Value	Effect Size (*r*)
Coffin	18.00 (17.00–19.00)	27.55 (24.88–30.15)	10.50	*p* < 0.001	0.669
Pastern	18.50 (17.40–19.80)	26.55 (24.38–28.97)	61.50	*p* < 0.001	0.657
Fetlock	18.65 (18.00–20.80)	26.70 (24.75–28.30)	81.50	*p* < 0.001	0.652
MLT	18.47 (17.73–19.70)	27.12 (25.11–28.82)	6.50	*p* < 0.001	0.670

Data are presented as median and interquartile range (IQR). Differences between forelimbs with and without pathology were assessed using the Mann–Whitney U test. Effect size is reported as r.

**Table 4:** Comparison of thermographic limb temperature indicators (°C) between pathological and non-pathological hindlimbs.

Thermographic Measurements	No Pathology (*n* = 61)	Pathology (*n* = 133)	U	*p*-Value	Effect Size (*r*)
Coffin	19.10 (18.15–19.75)	30.30 (28.50–31.60)	0.00	*p* < 0.001	0.797
Pastern	19.80 (19.00–20.80)	29.10 (26.90–30.00)	12.00	*p* < 0.001	0.795
Fetlock	19.50 (18.80–20.40)	28.20 (27.00–29.20)	1.00	*p* < 0.001	0.797
MLT	19.47 (19.00–20.16)	29.00 (27.27–30.10)	0.00	*p* < 0.001	0.797

Data are presented as median and interquartile range (IQR). Differences between hindlimbs with and without pathology were assessed using the Mann–Whitney U test. Effect size is reported as r.

**Table 5:** Thermographic measurements in healthy forelimbs, infectious lesions, and claw horn/mechanical lesions (*n* = 194 forelimbs).

Thermographic Measurements	Healthy (*n* = 158)	Mechanical (*n* = 30)	Infectious (*n* = 6)	H	*p*	ε^2^
Coffin	18.00 (17.00–19.00) ^a^	27.55 (24.80–30.68) ^b^	27.65 (27.42–28.70) ^b^	86.91	<0.001	0.445
Pastern	18.50 (17.40–19.80) ^a^	26.50 (23.85–29.28) ^b^	26.85 (26.20–27.35) ^b^	83.87	<0.001	0.429
Fetlock	18.65 (18.00–20.80) ^a^	26.10 (24.45–28.20) ^b^	27.85 (26.80–28.38) ^b^	82.77	<0.001	0.423
MLT	18.47 (17.73–19.70) ^a^	26.85 (25.04–28.92) ^b^	27.50 (26.93–27.92) ^b^	87.13	<0.001	0.446

Data are presented as median (interquartile range, IQR). H denotes the Kruskal–Wallis test statistic, and ε^2^ (epsilon squared) was used to estimate effect size. Different superscript letters within a row indicate significant differences between groups according to Dunn’s post hoc test with Bonferroni correction (*p* < 0.05). According to commonly used interpretation thresholds, ε^2^ values > 0.14 indicate a large effect.

**Table 6:** Thermographic measurements in healthy hindlimbs, infectious lesions, and claw horn/mechanical lesions (*n* = 194 hindlimbs).

Thermographic Measurements	Healthy (*n* = 59)	Mechanical (*n* = 104)	Infectious (*n* = 29)	H	*p*	ε^2^
Coffin	19.10 (18.15–19.75) ᵃ	30.05 (28.00–31.30) ᵇ	30.80 (29.30–31.80) ᵇ	123.73	<0.001	0.644
Pastern	19.80 (19.00–20.80) ᵃ	28.60 (26.68–29.80) ᵇ	29.80 (28.70–30.30) ᶜ	126.23	<0.001	0.657
Fetlock	19.50 (18.80–20.40) ᵃ	27.95 (26.48–29.02) ᵇ	28.50 (27.70–29.70) ᶜ	124.46	<0.001	0.648
MLT	19.47 (19.00–20.16) ᵃ	28.68 (26.82–29.85) ᵇ	29.73 (28.70–30.40) ᶜ	126.33	<0.001	0.658

Data are presented as median (interquartile range, IQR). H denotes the Kruskal–Wallis test statistic, and ε^2^ (epsilon squared) was used to estimate effect size. Different superscript letters within a row indicate significant differences between groups according to Dunn’s post hoc test with Bonferroni correction (*p* < 0.05). According to commonly used interpretation thresholds, ε^2^ values > 0.14 indicate a large effect.

**Table 7:** Spearman correlation between teat surface temperature and milk composition parameters (*n* = 97 cows).

Milk Parameters	Spearman r	*p*-Value
Fat	−0.012	0.907
Protein	−0.219	0.031
Lactose	−0.059	0.566
SCC	0.030	0.770
Urea	−0.234	0.021

**Table 8:** Mixed-effects logistic regression model fSor prediction of CMT-positive quarters based on teat surface temperature, parity, and days in milk (DIM) (*n* = 388 udder quarters from 97 cows).

Variable	Coefficient (β)	SE	z	*p*-Value	Odds Ratio (OR)	95% CI for OR
Intercept	−49.15	12.65	−3.89	<0.001	—	—
Temperature	1.67	0.43	3.88	<0.001	5.30	2.28–12.31
Parity	0.02	0.17	0.10	0.923	1.02	0.72–1.43
DIM	−0.001	0.002	−0.50	0.617	1.00	0.995–1.003

**Table 9:** Interpretation of Thermographic diagnostic zones for mastitis detection.

Temperature Zone	Interpretation	Probability of Mastitis
<27 °C	Healthy udder tissue	Very low
27–29.5 °C	Suspicious/monitoring recommended	Intermediate
≥29.5 °C	Mastitis likely	Elevated

**Table 10:** Partial correlations between limb pathology and teat surface temperature after adjustment for milk composition parameters (*n* = 97 cows).

Quarter	Forelimb Pathology (Partial r)	*p*-Value	Hindlimb Pathology (Partial r)	*p*-Value
RF	0.065	0.367	0.235	0.001
LF	0.132	0.066	0.232	0.001
RR	0.054	0.454	0.260	<0.001
LR	0.068	0.343	0.262	<0.001

## Data Availability

The data presented in this study are available on request from the corresponding author.
